# Synthesis of ((CeO_2_)_0.8_(Sm_2_O_3_)_0.2_)@NiO Core-Shell Type Nanostructures and Microextrusion Printing of a Composite Anode Based on Them

**DOI:** 10.3390/ma15248918

**Published:** 2022-12-13

**Authors:** Tatiana L. Simonenko, Nikolay P. Simonenko, Philipp Yu. Gorobtsov, Oleg Yu. Grafov, Elizaveta P. Simonenko, Nikolay T. Kuznetsov

**Affiliations:** 1 Kurnakov Institute of General and Inorganic Chemistry of the Russian Academy of Sciences, 31 Leninsky pr., Moscow 119991, Russia; 2 A. N. Frumkin Institute of Physical Chemistry and Electrochemistry of the Russian Academy of Sciences, 31 Leninskiy pr., Moscow 119071, Russia

**Keywords:** hydrothermal synthesis, SDC, NiO, composite anode, core-shell, hierarchical structures, nanospheres, nanosheets, microextrusion printing, SOFC

## Abstract

The process of the hydrothermal synthesis of hierarchically organized nanomaterials with the core-shell structure with the composition ((CeO_2_)_0.8_(Sm_2_O_3_)_0.2_)@NiO was studied, and the prospects for their application in the formation of planar composite structures using microextrusion printing were shown. The hydrothermal synthesis conditions of the (CeO_2_)_0.8_(Sm_2_O_3_)_0.2_ nanospheres were determined, and the approach to their surface modification by growing the NiO shell with the formation of core-shell structures equally distributed between the larger nickel(II) oxide nanosheets was developed. The resulting nanopowder was used as a functional ink component in the microextrusion printing of the corresponding composite coating. The microstructure of the powders and the oxide coating was studied by scanning (SEM) and transmission electron microscopy (TEM), the crystal structure was explored by X-ray diffraction analysis (XRD), the set of functional groups in the powders was studied by Fourier-transform infrared spectroscopy (FTIR) spectroscopy, and their thermal behavior in an air flow by synchronous thermal analysis (TGA/DSC). The electronic state of the chemical elements in the resulting coating was studied using X-ray photoelectron spectroscopy (XPS). The surface topography and local electrophysical properties of the composite coating were studied using atomic force microscopy (AFM) and Kelvin probe force microscopy (KPFM). Using impedance spectroscopy, the temperature dependence of the specific electrical conductivity of the obtained composite coating was estimated.

## 1. Introduction

Today, meeting the rapidly growing global demand for affordable electricity as well as reducing the ecological impact, primarily in the context of reducing greenhouse gas emissions into the atmosphere, requires a significant restructuring of the existing energy sector and the development of the alternative energy sources. In this regard, the efforts of scientists and engineers in different countries have focused on the design of efficient, reliable, and environmentally friendly renewable energy sources, which could partially, and then completely, replace the traditional energy, which operates at the expense of fossil fuels [1]. A significant interest in this regard are electrochemical generators of energy, one rapidly developing representative of which is solid oxide fuel cells (SOFCs). Devices of this type enable the efficient electrochemical conversion (up to 80% or more efficiency) of fuel energy into electrical energy, bypassing the low-performance stage of its direct combustion, resulting in an improved environmental friendliness of the energy generation process [2,3]. The undoubted advantages of SOFCs are the wide range of fuel types allowed for use (hydrogen of different purity, various hydrocarbons, biofuel) as well as the lack of need for expensive catalysts based on noble metals compared to fuel cells of other types [4,5].

The conventional design of SOFCs is represented by two porous ceramic electrodes (cathode and anode), between which there is a layer of dense solid electrolyte [6]. At the same time, the anode layer plays an important role in the process of the entire device operation, providing the electrocatalytic oxidation of the fuel used [7]. In this regard, the key characteristics that should be met by an effective anode material of SOFCs are its high ionic and electronic conductivity, catalytic activity during the hydrogen oxidation reaction (HOR), and a developed pore structure that promotes an increase in the active centers involved in the HOR [8,9]. In addition, when selecting the anode material, it is necessary to take into account that its linear thermal expansion coefficient value should be high enough and correspond to similar values of other functional layers of the fuel cell when heating it in the operating temperature range of 500–800 °C [10]. Among the most popular and widely used anode materials for solid oxide fuel cells are composites based on Ni or NiO as well as a solid electrolyte (e.g., NiO-SDC, NiO-YSZ, Ni-GDC, etc. [11,12]), which allows combining the high catalytic activity of nickel (or its oxide) with an opportunity to increase the compatibility of the thermophysical characteristics of the anode and electrolyte. According to a number of authors, nickel-containing SOFC anodes are preferable to nickel-free ones, since they allow for efficient conversion of the hydrocarbon fuel (in particular, methane) directly in the fuel cell chamber and achieve higher power densities of the resulting fuel cell in the temperature range of 500–600 °C [6,10]. In this context, anode materials based on nickel oxide and cerium dioxide doped with samarium (NiO–SDC), which have significant advantages over the classical cermets based on yttrium-stabilized zirconium oxide (Ni-YSZ), have attracted considerable attention. These advantages are, in particular, attributed to the solid solutions in the CeO_2_-Sm_2_O_3_ system demonstrating higher values of ionic conductivity, lower activation energy values, and the highest stability in an atmosphere with low oxygen partial pressure in the intermediate temperature range compared to the analogues, which is due to the highest concentration of oxygen vacancies and the lowest value of the enthalpy of cation-vacancy association [13]. NiO–SDC anodes also demonstrate a higher chemical resistance to carbonization when using methane and other hydrocarbons as fuel [14,15,16,17,18].

The working characteristics of anodes are largely determined by their microstructure. Thus, it was shown that the use of highly dispersed initial powders and the achievement of a high degree of component mixing (in particular, nickel oxide and electrolyte particles) promote the formation of a material with a developed pore structure and a catalytically active surface [19,20]. The morphology and degree of phase distribution uniformity during mixing of the anode material components, in turn, largely depend on the synthesis methods used [1]. It was shown that the mechanical mixing of components does not make it possible to achieve the desired homogeneity of the material [10,21], whereas more complicated methods such as the co-precipitation of metal hydroxides and oxalates [22,23], combustion methods [24,25] or spray pyrolysis [26,27] offer more possibilities to control the features of the microstructure of the produced anode and provide the formation of a more developed surface. In this work, in order to form an anode material with the core-shell structure of the composition ((CeO_2_)_0.8_(Sm_2_O_3_)_0.2_)@NiO, we propose the use of a hydrothermal method that makes it possible to obtain nanomaterials with the given type of microstructure including the hierarchical organization of particles due the control of a wide range of synthesis parameters (temperature, reactor pressure, duration of hydrothermal treatment, concentration and type of reagents, etc.) [7,28,29,30]. In addition, this method allows for the formation of materials with a core-shell type structure, which can contribute to a better contact of oxide particles of different chemical compositions as well as their more uniform distribution in the structure of the anode material.

Recently, the trend toward the miniaturization of microelectronic and alternative energy devices has become more and more prominent. For example, there are a number of works on the formation of micro-SOFCs for a variety of portable applications [31,32]. The formation of planar miniaturized nanostructures, which additionally contribute to the decrease in the fuel cell resistance and enhance its power, requires the involvement of specialized technologies that ensure the addressability of material deposition, reproducibility, and specified geometric parameters of the formed structures. Standard methods of photolithography as well as sputtering technologies (CVD, plasma, or laser sputtering) are not effective in this case, since it is difficult to obtain materials with a complex chemical composition as well as control the level of porosity of the films obtained [33,34]. According to these studies [35,36], the use of standard technologies employed to create planar SOFCs—tape casting and screen printing—does not allow the formation of electrode structures with a width below 0.5 mm and a gap between them of less than 0.2 mm. An effective solution to this problem is the use of additive technologies [37,38] to form electrode nanostructures with the required geometry and microstructure. One of the most promising approaches in this case is microextrusion printing (also known as direct writing) [39,40,41,42], which allows for the formation of complex geometry patterns using functional inks characterized by a wide range of acceptable viscosities (from 30 to 6⋅10^7^ mPa⋅s [43]).

Therefore, we studied the hydrothermal synthesis of hierarchically organized nanomaterials with the core-shell structure with the composition ((CeO_2_)_0.8_(Sm_2_O_3_)_0.2_)@NiO, and show the prospects for their application in the formation of composite planar anodic structures by microextrusion printing.

## 2. Materials and Methods

### 2.1. Chemicals

Ce(NO_3_)_3_·6H_2_O (99.99%, LANHIT, Moscow, Russia), Sm(NO_3_)_3_·6H_2_O (99.99%, LANHIT, Moscow, Russia), NiCl_2_·6H_2_O (>98%, RUSHIM, Moscow, Russia), (NH_2_)_2_CO (99%, RUSHIM, Moscow, Russia), α-terpineol (>97%, Vekton, St. Petersburg, Russia), ethyl cellulose (48.0–49.5% (*w/w*) ethoxyl basis, Sigma Aldrich, St. Louis, MO, USA) were used in this work without further purification.

### 2.2. Preparation of ((CeO_2_)_0.8_(Sm_2_O_3_)_0.2_)@NiO Core-Shell Structures 

The formation of ((CeO_2_)_0.8_(Sm_2_O_3_)_0.2_)@NiO composite nanopowders of core-shell type with the hierarchical organization of particles was performed using the hydrothermal method. In the first stage, an aqueous solution (60 mL in volume) of cerium and samarium nitrates mixed in a given stoichiometric ratio was prepared (the total concentration of metals was 0.01 mol/L). After that, 0.36 g of urea was added to the obtained solution under continuous stirring and the obtained reaction system was transferred into a 100 mL Teflon-lined stainless-steel autoclave and heated in a muffle furnace to 120 °C at a rate of 1.5°/min, followed by an exposure time at this temperature for 1 h. After heat treatment, the reaction system was cooled naturally with the furnace to a temperature of 25 °C. After cooling, the dispersed phase was separated from the dispersion medium by cyclic centrifugation, washed with distilled water, and then subjected to drying at 100 °C (3 h) and additional heat treatment (600 °C, 1 h) to form an oxide composition (CeO_2_)_0.8_(Sm_2_O_3_)_0.2_.

At the next stage, the hydrothermal growth of the nickel oxide shell was carried out on the surface of the particles of the obtained ((CeO_2_)_0.8_(Sm_2_O_3_)_0.2_) powder. For this purpose, a 60 mL reaction system was prepared based on an aqueous solution of nickel chloride (c = 6.5·10^−4^ mol/L) and 0.082 g of urea. Then, a previously obtained powder with a (CeO_2_)_0.8_(Sm_2_O_3_)_0.2_ composition was added under stirring to the reaction system, after which it was placed in a similar autoclave and hydrothermally treated at 160 °C for 1.5 h (the heating rate was also 1.5°/min). After natural cooling of the reactor, the dispersed phase was separated from the mother liquor by cyclic centrifugation and washed with distilled water, which was then dried (100 °C, 3 h) and further heat treated (600 °C, 2 h) to crystallize the nickel oxide and form the target material with a core-shell structure with the composition ((CeO_2_)_0.8_(Sm_2_O_3_)_0.2_)@NiO.

### 2.3. Microextrusion Printing of ((CeO_2_)_0.8_(Sm_2_O_3_)_0.2_)@NiO Composite Anode 

The obtained nanopowder of the composition ((CeO_2_)_0.8_(Sm_2_O_3_)_0.2_)@NiO was further used in the preparation of functional inks for microextrusion printing of the coating with the corresponding composition. As a solvent, α-terpineol was used, and ethyl cellulose was employed as a binder and pore forming agent. The mass fraction of the solid phase in the composition of functional inks was 25%. Microextrusion printing of the composite anode coating was performed on the surface of specialized Pt/Al_2_O_3_/Pt chips (R_a_ = 100 nm, geometric dimensions 4.1 × 25.5 × 0.6 mm), according to the procedure we described earlier [41,42]. After the printing process was completed, the coating was dried (70 °C, 24 h) and further heat treated (600 °C, 1 h) to remove the residual solvent and to decompose the organic constituents included in the functional inks.

### 2.4. Investigation of the Materials Obtained

Thermal properties of the obtained powders before and after application of the NiO shell were studied by simultaneous (TGA/DSC) thermal analysis (SDT Q-600 thermal analyzer, TA Instruments, New Castle, DE, USA) in an air flow in the temperature range of 25–1000 °C (controlled heating was carried out in Al_2_O_3_-crucibles at a rate of 10°/min in an air current, the gas flow rate was 250 mL/min).

In order to record the infrared transmission spectra of powders, the corresponding suspensions were prepared in Vaseline oil, which were then placed between the glasses of potassium bromide as films. Spectra were recorded in the 350–4000 cm^−1^ wavenumber range (signal accumulation time was 15 s and resolution was 1 cm^−1^) using an InfraLUM FT-08 FTIR spectrometer (Lumex, St. Petersburg, Russia).

X-ray diffraction analysis (XRD) of the obtained powders was carried out with a Bruker D8 Advance diffractometer (Bruker, Bremen, Germany; CuK_α_ = 1.5418 Å, Ni-filter, E = 40 keV, I = 40 mA, 2θ range—15°–80°, resolution—0.02°, signal accumulation time was 0.5 s (for powders) and 2.0 s (for coating analysis)). Analysis of the XRD spectra was carried out with the use of the Rietveld refinement method, implemented in X’Pert HighScore Plus software (PANalytical B.V., Almelo, The Netherlands). The d-spacing (interplanar spacing) of each plane was calculated using Bragg’s equation:

(1) 2dsinθ=nλ,

where *n* is the diffraction order; λ is the X-ray wavelength; *d* is the interplanar spacing; and θ is the Bragg’s angle.

X-ray photoelectron spectroscopy (XPS) studies were performed using an OMICRON ESCA + spectrometer (Scienta Omicron, Uppsala, Sweden) with an aluminum anode equipped with an AlKα XM1000 monochromatic X-ray source (with an emission energy of 1486.6 eV and a power of 252 W). A CN-10 charge neutralizer with an emission current of 4 μA and a beam energy of 1 eV was used to eliminate the local charge on the analyzed surface. The transmittance energy of the analyzer was 20 eV for the high resolution spectra of single elements. The spectrometer was calibrated using the Au4f 7/2 line at 84.1 eV. The pressure in the analyzer chamber did not exceed 10^−9^ mbar. All spectra were accumulated at least eight times. Fluctuation of the peak positions did not exceed ±0.1 eV.

The surface microstructure of the obtained oxide powders was studied by scanning (SEM; Carl Zeiss NVision-40, Carl Zeiss, Inc., Oberkochen, Germany) and transmission electron microscopy (TEM; JEOL JEM-1011 (JEOL Ltd., Akishima, Tokyo, Japan) with an ORIUS SC1000W digital camera (Gatan Inc., Pleasanton, CA, USA).

The resulting oxide coating was also studied by atomic force microscopy (AFM) including Kelvin probe force microscopy (KPFM). The NT-MDT Solver PRO microscope (NT-MDT, Zelenograd, Russia) and ETALON HA_HR probes (ScanSens, Bremen, Germany) with a W_2_C conductive coating (rounding radius < 35 nm) were used for this purpose. The electronic work function of the materials’ surface (ϕ_film_) was determined during the KPFM measurement. For this purpose, the film surface was scanned with a probe with a known electronic work function (ϕ_tip_), the average value of the contact potential (ϕ_CPD_) was calculated for the scanned area, and then ϕ_film_ was determined as the difference between ϕ_tip_ and ϕ_CPD_. A volt–ampere characteristic for a single point on the coating surface was also recorded using the atomic force microscope. For this purpose, the microscope was switched to the contact mode of scanning, and the current flowing between the probe and the sample was measured when the bias voltage was applied. The bias voltage was applied through a metal clamp contacting the platinum onto the Pt/Al_2_O_3_/Pt chip.

The study of the electrical conductivity of the thick film of the composition ((CeO_2_)_0.8_(Sm_2_O_3_)_0.2_)@NiO, printed on the surface of the Pt/Al_2_O_3_/Pt chips, was carried out by impedance spectroscopy using a professional electrochemical workstation based on potentiostat/halvanostat P-45X with an FRA-24M impedance measurement module (Electrochemical Instruments, Chernogolovka, Russia). The impedance hodographs were recorded in the frequency range of 1 MHz-1 Hz in the air in the temperature range of 50–600 °C. The temperature of the sample was maintained by feeding a voltage (power supply QJ 3003H) to that previously applied on the reverse side of the chip platinum microheater and was controlled by a thermal imager Testo 868. The value of the electrical resistance of the composite anode ((CeO_2_)_0.8_(Sm_2_O_3_)_0.2_)@NiO was calculated from the electrochemical impedance spectroscopy data using ZView software (Version 3.3c, Scribner Associates Inc., Southern Pines, NC, USA).

## 3. Results and Discussion

**Figure 1:**
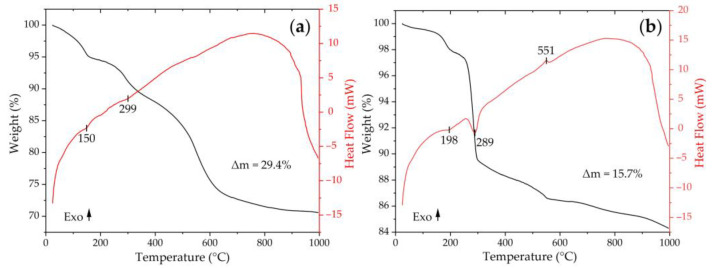
TGA (black) and DSC (red) curves for semiproducts obtained in the hydrothermal synthesis of (CeO_2_)_0.8_(Sm_2_O_3_)_0.2_ oxide (**a**) and after applying the nickel oxide shell on its surface (**b**).

First, the thermal behavior of the semiproduct obtained by the hydrothermal synthesis of the (CeO_2_)_0.8_(Sm_2_O_3_)_0.2_) oxide was studied (Figure 1a). As can be seen from the thermograms, when the powder was heated up to 1000 °C, a 4-step weight loss took place in the temperature ranges of 25–160 (Δm = 5.1%), 160–340 (5.4%), 340–650 (16.1%), and 650–1000 °C (2.8%), and the total weight loss was 29.4%. The first step, accompanied by a weak endothermic effect, was associated with the evaporation of the residual solvent, and the desorption of atmospheric gases and water molecules. At the next stage, partial decomposition of the semiproduct occurred (most likely chemically bound OH-groups are removed), after which this process intensified, resulting in a sharp increase in mass loss (at this stage, presumably, decomposition of other functional groups—in particular, carbonate ions—occurs). A further increase in temperature showed a significant slowing of the mass loss. Thus, the results of synchronous thermal analysis suggest that cerium-samarium hydroxocarbonate was obtained as a semiproduct, and the main processes of its thermal transformation into the oxide of the composition (CeO_2_)_0.8_(Sm_2_O_3_)_0.2_ were completed before 600 °C, which allowed us to identify the optimal mode of the additional heat treatment of the material (600 °C, 1 h), in order to obtain an oxide powder of the target composition. 

A composite semiproduct with a core-shell structure obtained after modification of the surface of the (CeO_2_)_0.8_(Sm_2_O_3_)_0.2_ particles with nickel oxide was also studied using thermal analysis (Figure 1b). It was assumed that during hydrothermal synthesis, a nickel hydroxide shell is formed, the thermal transformations of which are shown in the corresponding thermograms. Thus, in this case, a 5-step mass loss was observed on the TGA curve: 25–130 (Δm = 0.4%), 130–220 (1.8%), 220–300 (8.3%), 300–555 (3.0%), and 555–1000 °C (2.2%) in contrast to the previous case. The total weight loss over the entire temperature range under study was 15.7%. During the first stage, the mass decrease was due to the evaporation of the residual solvent as well as the desorption of atmospheric gases and water molecules. Further heating led to a 2-step mass loss accompanied by endothermic effects and associated with the decomposition of nickel hydroxide. In the fourth stage, the continuing decomposition of the semiproduct was probably combined with the crystallization of nickel oxide, accompanied by exothermic effects with a maximum at 551 °C. In the last step, in the high-temperature interval, the processes of semiproduct decomposition are completed. From the thermal analysis results, it can be seen that, as in the previous case, the main processes of thermal transformation of the semiproduct and formation of the NiO shell were finished before 600 °C. Thus, the optimal mode of the composite semiproduct with additional heat treatment (600 °C, 1 h) was determined.

**Figure 2:**
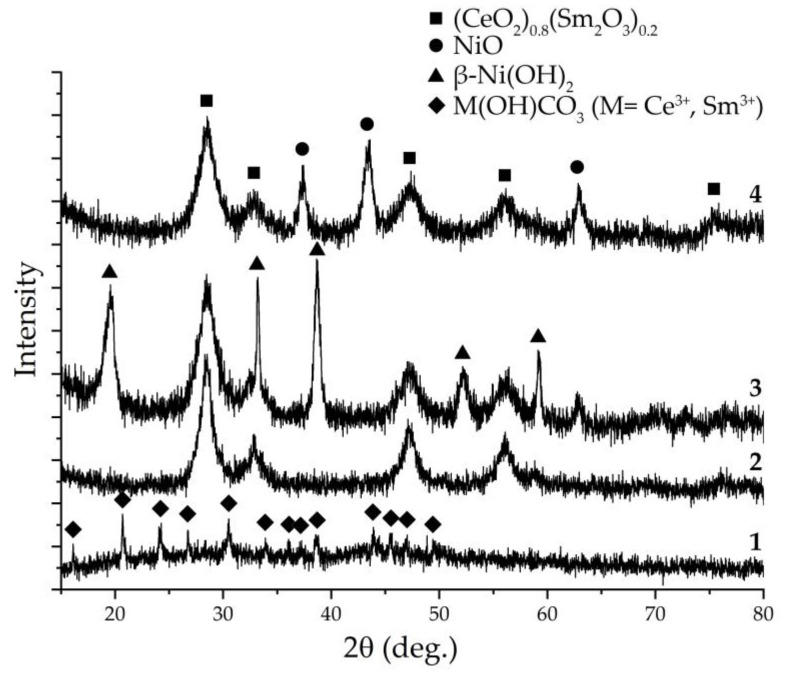
X-ray diffraction patterns of M(OH)CO_3_ (M = Ce^4+^; Sm^3+^) (**1**), (CeO_2_)_0.8_(Sm_2_O_3_)_0.2_ (**2**), ((CeO_2_)_0.8_(Sm_2_O_3_)_0.2_)@β-Ni(OH)_2_ (**3**), and ((CeO_2_)_0.8_(Sm_2_O_3_)_0.2_)@NiO (**4**) powders after drying (**1** and **3**) and additional heat treatment (**2** and **4**).

The crystal structure of the semiproducts and powders subjected to additional heat treatment was examined by X-ray diffraction analysis (Figure 2). As can be seen from the X-ray diffraction pattern of the semiproduct formed during the synthesis of (CeO_2_)_0.8_(Sm_2_O_3_)_0.2_ (Figure 2, sample 1), there is a set of reflexes corresponding to the orthorhombic structure (sp.gr *Pnma*), typical for cerium and samarium hydroxocarbonates, which agrees well with the results of synchronous thermal analysis. Calcination of this powder at 600 °C (1 h) led to complete decomposition of the semiproduct, which was confirmed by the absence of the corresponding reflexes, and the formation of a (CeO_2_)_0.8_(Sm_2_O_3_)_0.2_ solid solution with a fluorite-type structure (sp.gr *Fm3m*) with an average coherent scattering region (CSR) size of about 8.0 ± 0.7 nm (Figure 2, sample 2). The analysis of the semiproduct crystal structure obtained after the NiO shell formation and drying at 100 °C (Figure 2, sample 3) indicates the appearance of a composite powder based on a core ((CeO_2_)_0.8_(Sm_2_O_3_)_0.2_ solid solution) and a shell representing the hexagonal β-Ni(OH)_2_ phase (sp.gr *P-3m1*), which is consistent with thermal analysis data. Nickel hydroxide decomposes into a cubic NiO structure (sp.gr. Fm3m) upon additional heat treatment at 600 °C, resulting in a ((CeO_2_)_0.8_(Sm_2_O_3_)_0.2_)@NiO core-shell composite powder (Figure 2, sample 4). The average CSR size for samarium doped ceria in this case was 5.1 ± 0.5 nm, and for nickel(II) oxide, this parameter had a value of 9.7 ± 0.8 nm. Therefore, during the formation of the NiO shell, the average CSR size of the (CeO_2_)_0.8_(Sm_2_O_3_)_0.2_ nanopowder decreased by 36% due to hydrothermal treatment in the nickel-containing reaction system. Thus, the X-ray diffraction analysis confirmed the formation of a nanoscale composite powder with the composition of ((CeO_2_)_0.8_(Sm_2_O_3_)_0.2_)@NiO that did not contain any crystalline impurities. According to Bragg’s Equation (1), the interplanar spacings (d, Å) were calculated for each of the composite powder components: (CeO_2_)_0.8_(Sm_2_O_3_)_0.2_—3.130 (111), 2.732 (200), 1.924 (220), 1.639 (311), and 1.258 (331). The crystal lattice parameter *a* for this solid solution was 5.464 Å, which agrees well with the literature data (ICDD PDF no. 01-075-0158). For the NiO shell, the calculated values of the interplanar spacings were 2.407 (111), 2.085 (200), and 1.476 Å (220). At the same time, the value of the *a* parameter of the nickel(II) oxide crystal lattice had a value of 4.189 Å, which also agrees reasonably well with the literature (ICDD PDF no. 01-073-1523).

**Figure 3:**
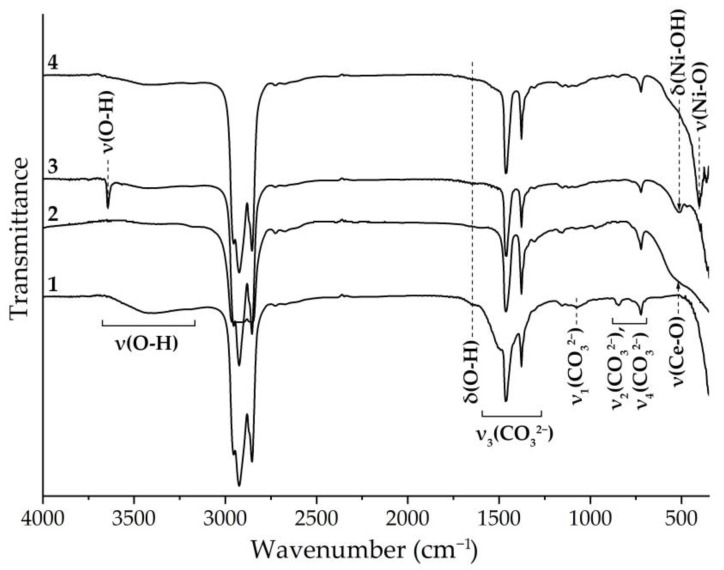
IR spectra of the M(OH)CO_3_ (M = Ce^4+^; Sm^3+^) (**1**), (CeO_2_)_0.8_(Sm_2_O_3_)_0.2_ (**2**), ((CeO_2_)_0.8_(Sm_2_O_3_)_0.2_)@β-Ni(OH)_2_ (**3**), and ((CeO_2_)_0.8_(Sm_2_O_3_)_0.2_)@NiO (**4**) powders after drying (**1** and **3**) and additional heat treatment (**2** and **4**).

The semiproducts and oxide powders obtained during the synthesis of (CeO_2_)_0.8_(Sm_2_O_3_)_0.2_ and after growing the NiO shell on its surface were studied by FTIR spectroscopy, where the results allowed us to confirm the data of the X-ray phase analysis. It can be seen that after hydrothermal treatment of the reaction system containing cerium and samarium nitrates as well as urine, the formation of cerium and samarium hydroxocarbonates takes place, which is indicated by characteristic absorption bands within the wavenumber intervals of 3100–3700 and 1620–1680 cm^−1^ related, respectively, to the stretching and bending vibrations of OH-groups, as well as absorption bands in the range of 1620–500 cm^−1^, referring to the vibrations of coordinated carbonate groups (Figure 3, spectrum 1). In particular, in the wavenumber range of 1620–1300 cm^−1^, there was an overlap of the Vaseline oil bands and the ν_3_ mode (antisymmetric stretching vibrations of C–O bonds within carbonate groups), while in the area of lower wavenumbers, there were also the ν_1_ (1095–1050 cm^−1^), ν_2_ (870–820 cm^−1^), and ν_4_ (780–690 cm^−1^) modes, caused by symmetrical stretching vibrations of the C–O bonds within carbonate groups as well as the deformation vibrations of carbonate anions out-of-plane and in-plane, respectively [42,44]. Additional heat treatment of the resulting powder (600 °C, 1 h), as seen in spectrum 2, led to a significant decrease in the intensity of absorption bands, corresponding to the vibrations of OH-bonds. Furthermore, the absorption bands referring to carbonate group vibrations were absent in the above spectrum, which indicates the complete decomposition of the hydroxocarbonates and oxide material formation, as indicated by the appearance of a shoulder in the 650–430 cm^−1^ interval, characteristic of the Ce–O stretching vibrations.

Subsequent treatment of the obtained (CeO_2_)_0.8_(Sm_2_O_3_)_0.2_ powder in a reaction system containing nickel chloride and urea led to the formation of β-nickel(II) hydroxide on the surface of the oxide particles (Figure 2, spectrum 3). This is primarily indicated by the absorption bands with maximums of about 1640 and 3650 cm^−1^ referring to the vibrations of the OH- groups as well as the characteristic band with a maximum of 515 cm^−1^ referring to bending vibrations of the Ni–OH bond [45]. Heat treatment of the obtained powder at 600 °C resulted in the decomposition of β-Ni(OH)_2_ and the formation of NiO on the surface of the (CeO_2_)_0.8_(Sm_2_O_3_)_0.2_ particles, which was accompanied by both the absence of the functional group absorption bands described above and by the appearance of a band due to Ni–O stretching vibrations in the range of 400–470 cm^−1^ (Figure 2, spectrum 4) [46].

**Figure 4:**
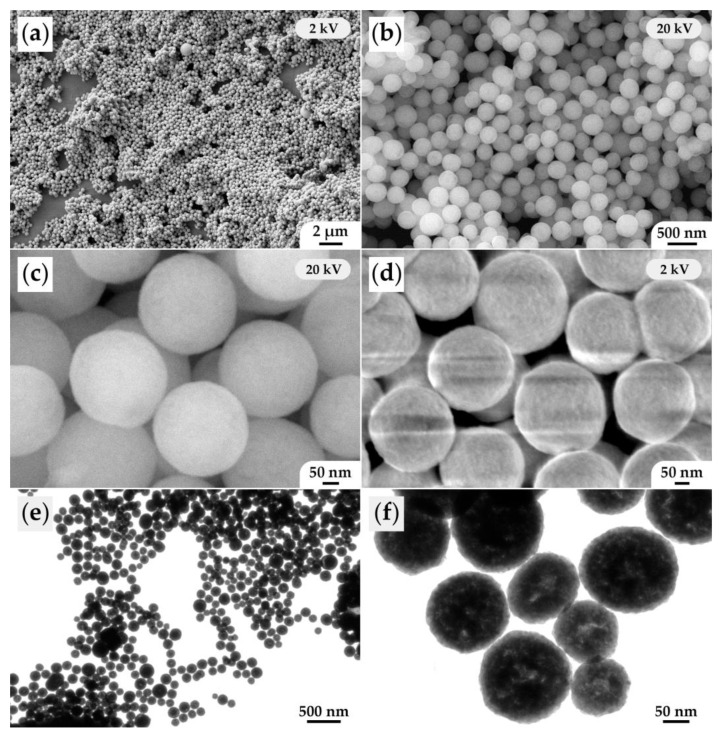
Microstructure of the obtained (CeO_2_)_0.8_(Sm_2_O_3_)_0.2_ powder according to the SEM (**a**–**d**) and TEM data (**e**,**f**).

The microstructure of the obtained (CeO_2_)_0.8_(Sm_2_O_3_)_0.2_ powder was analyzed by scanning (Figure 4a–d) and transmission electron microscopy (Figure 4e,f). As can be seen from the SEM results, the powder consists of nanospheres with a rather narrow size distribution, with an average size of about 250 nm. Investigation of the material surface at reduced accelerating voltage (Figure 4d) allowed us to determine that the nanospheres consisted of smaller particles, having an average size of 10 ± 2 nm, which agrees well with the average CSR size determined when the corresponding X-ray diffraction patterns were analyzed. According to the TEM data, the oxide nanospheres had inhomogeneous density—there were more transparent areas of the material for the electron beam, which is probably due to the specific mechanism of directional self-organization of the material in hydrothermal conditions. Moreover, one can also easily see that the nanospheres had a textured surface and were composed of smaller particles.

**Figure 5:**
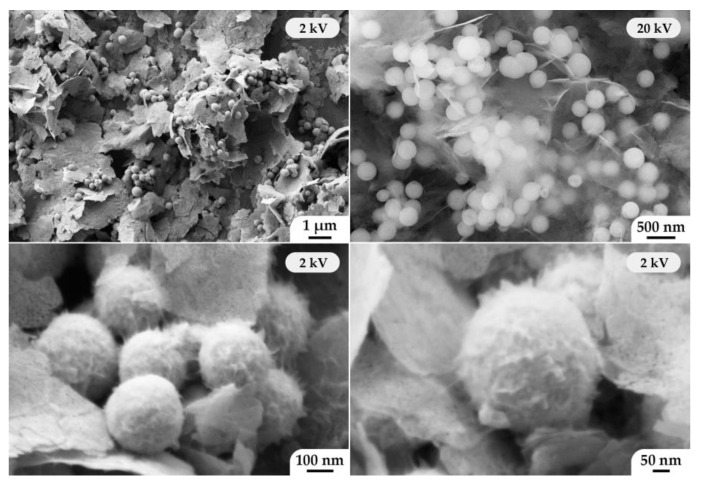
Microstructure of the obtained ((CeO_2_)_0.8_(Sm_2_O_3_)_0.2_)@NiO powder with the core-shell structure according to the SEM data.

The scanning electron microscopy helped to reveal a composite structure of the obtained ((CeO_2_)_0.8_(Sm_2_O_3_)_0.2_)@NiO powder (Figure 5). As seen in the micrographs, the (CeO_2_)_0.8_(Sm_2_O_3_)_0.2_ nanospheres and the formed NiO nanosheets (less than 10 nm thick) were evenly distributed among each other. Material examination at elevated accelerating voltage (20 kV) allows for an understanding of the material microstructure features to a greater depth due to the very insignificant thickness of NiO nanosheets (semitransparent in this analysis mode). Thus, one could observe that nanospheres of CeO_2_-based solid solution were located not only on the outer surface of the nickel(II) oxide nanosheets, but also in the volume of agglomerates on their basis. During the study of the oxide nanosphere microstructure, it was found that a shell of thin NiO nanosheets with a developed hierarchically organized surface formed on their surface under hydrothermal conditions. Hence, spherical core-shell nanostructures of the composition ((CeO_2_)_0.8_(Sm_2_O_3_)_0.2_)@NiO, evenly distributed between the larger nickel(II) oxide nanosheets, were formed during the synthesis process.

**Figure 6:**
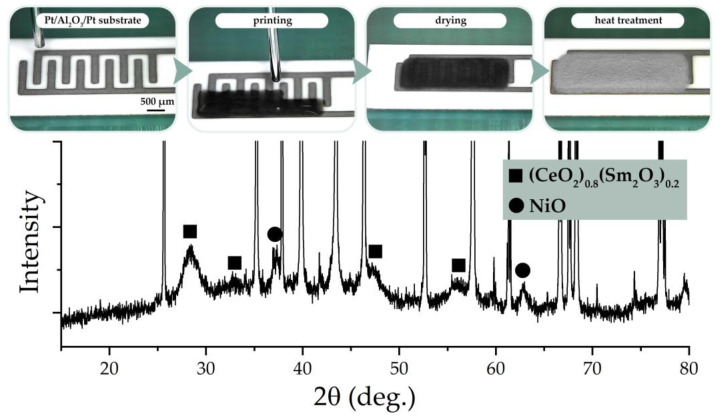
Microextrusion printing process of the ((CeO_2_)_0.8_(Sm_2_O_3_)_0.2_)@NiO composite anode film (**top**) and the corresponding X-ray diffraction pattern (**bottom**).

The crystal structure of the ((CeO_2_)_0.8_(Sm_2_O_3_)_0.2_)@NiO composite coating obtained according to the scheme (Figure 6, top) by microextrusion printing was also studied using X-ray diffraction analysis (Figure 6, bottom). As can be seen from the XRD pattern, against the background of intense narrow reflexes related to the substrate material (Pt/Al_2_O_3_/Pt chip), signals from the composite coating elements—(CeO_2_)_0.8_(Sm_2_O_3_)_0.2_ solid solution and NiO shell, whose crystal structure did not change during the microextrusion printing—were observed. The average CSR size of the samarium-doped cerium dioxide remained practically unchanged (4.9 ± 0.5 nm) in comparison with the composite powder used for the preparation of functional inks.

**Figure 7:**
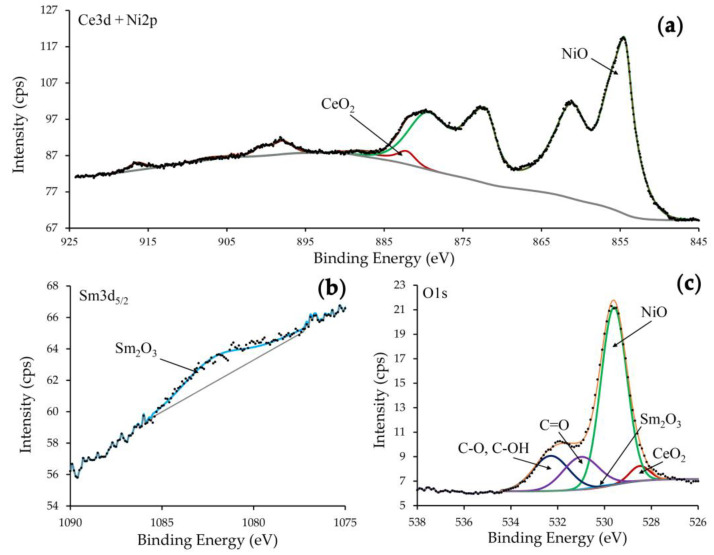
High-resolution (Ce3d + Ni2p) (**a**), Sm3d_5/2_ (**b**), and O1s (**c**) XPS spectra of the printed ((CeO_2_)_0.8_(Sm_2_O_3_)_0.2_)@NiO composite anode film.

The surface of the obtained coating was analyzed by X-ray photoelectron spectroscopy (Figure 7). The Ce3d and Ni2p region spectra (Figure 7a) showed that the nickel curve exhibited characteristic peaks with the positions of the Ni2p_3/2_-Ni2p_1/2_ spin-orbit splitting maxima at 854.7 and 872.5 eV, respectively, as well as prominent satellite peaks at 861.4 and 879.5 eV. The asymmetry and the large satellite fraction of the Ni2p_3/2_-component allowed us to correlate nickel with the NiO state. The Ce3d_5/2_ component overlapped the nickel region, however, the presence of a peak at 916.4 eV as well as the peaks typical of Ce(IV) in the 887–922 eV range suggest its chemical state as CeO_2_. Due to the low amount of samarium in the composite structure under study, the signal accumulation was performed at the Sm3d_5/2_ region (Figure 7b). This spectrum showed a low-intensity, asymmetric peak at 1082.6 eV in the region of lower binding energies. The complex oxygen O1s spectrum can be described by five peaks, three of which refer to CeO_2_ (528.5 eV), NiO (529.5 eV), and Sm_2_O_3_ with a maximum at 530.2 eV, and the other two belong to oxygen-containing carbon impurities: C–O, C–OH (532.2 eV), and C=O at 531 eV (Figure 7c).

**Figure 8:**
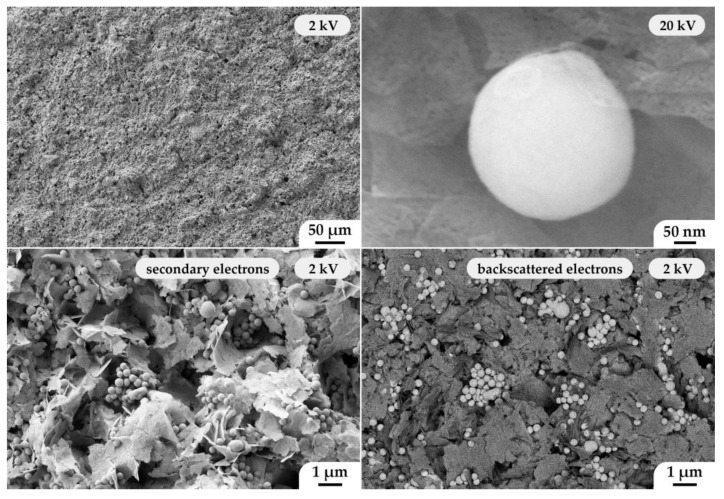
Microstructure of a planar composite anode of composition ((CeO_2_)_0.8_(Sm_2_O_3_)_0.2_)@NiO according to the SEM data.

Using scanning electron microscopy, the microstructure of the printed composite coating was also studied (Figure 8). The material was characterized by high porosity, which is considered to be one of the most important characteristics of anodic coatings. At the same time, its microstructure fully corresponds to the composite powder employed. Thus, modified nanospheres with a core-shell structure with a composition ((CeO_2_)_0.8_(Sm_2_O_3_)_0.2_)@NiO were evenly distributed between the NiO nanosheets. Surface analysis of the corresponding coating area using a backscattered electron detector revealed a phase contrast between the modified solid electrolyte nanospheres and the nickel(II) oxide nanosheets. Investigation of the material surface using a secondary electron detector at an elevated accelerating voltage revealed that the NiO nanosheets were highly porous (the average pore size was 8 ± 1 nm), and their thickness, as in the case of the composite powder employed, did not exceed 10 nm. Thus, microextrusion printing makes it possible to preserve the microstructural features of the nanopowders used in the formation of the corresponding functional coatings.

**Figure 9:**
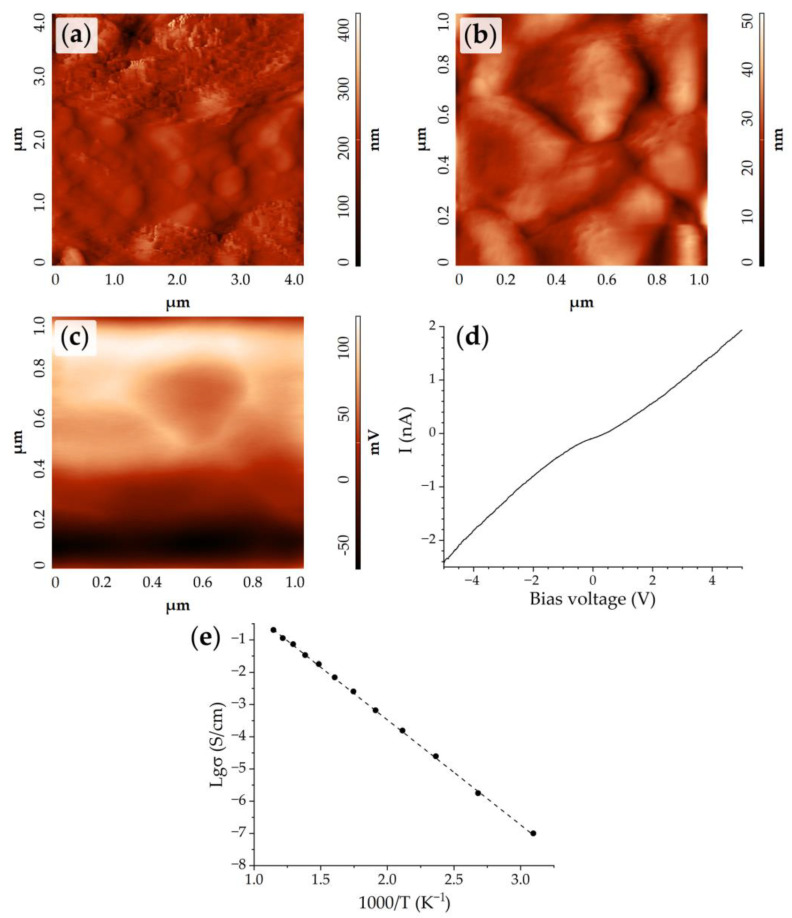
AFM results of the ((CeO_2_)_0.8_(Sm_2_O_3_)_0.2_)@NiO composite coating: topography (**a**,**b**), map of the surface potential distribution (the result of KPFM; (**c**)), typical volt–ampere curve for the characteristic local area on the coating surface (**d**). Temperature dependence of the film specific conductivity ((CeO_2_)_0.8_(Sm_2_O_3_)_0.2_)@NiO (**e**).

The surface morphology of the ((CeO_2_)_0.8_(Sm_2_O_3_)_0.2_)@NiO composite anode with the core-shell structure was also studied using AFM. The topographic images (Figure 9a,b) demonstrated the same structures in the SEM micrographs: nanosheets with high porosity formed from nano-particles (pore sizes were ~30–60 nm) and spheres with diameters of 300–400 nm. It can be seen that the spheres did not have a smooth surface, but were covered by a film of NiO with a developed surface. Upon examination of the composite coating morphology by the AFM method, its thickness was evaluated, measuring 30 μm.

The local electrophysical properties of the coating were estimated with the help of KPFM. The surface potential distribution map (Figure 9c) showed that the boundaries between the spheres were slightly lighter than their main surface. This indicates a shift in the density of charge carriers to the area of the grain boundaries. The work function (ϕ_film_) of the nanosphere surface according to the KPFM results was 4.698 eV. This was lower than the values of the work function for other NiO-based materials previously obtained: 4.751 eV [47], 5.125 eV [41], and 4.84 eV [48], which may be explained by the electrophysical interaction between the NiO shell and the core ((CeO_2_)_0.8_(Sm_2_O_3_)_0.2_)@NiO. The fact is that NiO is a p-type semiconductor, while CeO_2_ (including that doped with samarium) is the n-type. Thus, a p–n junction occurs at the boundary between the core and the shell, leading to the convergence of band edges and, thus, the convergence of the Fermi levels of the materials [49,50]. This is particularly characteristic of the depletion layer at the boundary between the two phases. Since in our case the NiO shell was thin, we can assume that the size of the depleted layer is at least comparable to the NiO oxide layer thickness, and thus the p–n junction affects the measured work function value. In these circumstances, the Fermi level for the nickel oxide should increase and the work function should decrease. Most likely, this explains the reduced value of the material’s work function in a particular case.

The volt–ampere characteristic of the material can be of interest in terms of detecting the presence of a noticeable p–n junction. We recorded such characteristics for a number of local areas on the composite anode surface (Figure 9d). It can be seen that the material, in general, had a sufficiently high conductivity for oxide films—the current strength reached several nanoamperes. A nonlinear dependence of current on the bias voltage over the entire voltage range could also be observed. This was due to the presence of several rectifying contacts in the resulting circuit: platinum–oxide film, oxide film–probe. At the same time, the volt–ampere characteristic was not symmetrical: the rectification ratio (RR) (ratio of the current at forward bias voltage to reverse bias voltage with the same value) was 0.67, however, if there are only two such rectifying contacts directed oppositely, one can expect a RR of about 1 [51]. This can indicate the presence of an additional rectifying contact associated with the p–n junction between the (CeO_2_)_0.8_(Sm_2_O_3_)_0.2_ core and the NiO shell.

In addition, the specific electrical conductivity of the obtained planar material was evaluated on the basis of the impedance spectroscopy data. The calculation of the activation energy of electrical conductivity was carried out by Formula (2):

(2) $$\sigma =\sigma_{0}\cdot e^{\frac{-Ea}{kT}},$$

where *σ* is the specific conductivity of the coating; *σ*_0_ is the pre-exponential factor; *k* is the Boltzmann constant; *E_a_* is the activation energy of conductivity.

The obtained temperature dependence of the specific conductivity of the material under study (Figure 9e) had a linear character; with an increase in temperature from 50 to 600 °C, the conductivity increased by several orders of magnitude, and the activation energy of conductivity was 0.64 eV. In the case of the formed composite material, both NiO and (CeO_2_)_0.8_(Sm_2_O_3_)_0.2_ contribute to the formation of the conductive net that provides the overall conductivity of the material. As a result, it was shown that the proposed approach based on the hydrothermal synthesis of a ((CeO_2_)_0.8_(Sm_2_O_3_)_0.2_)@NiO nanomaterial with a core-shell structure and the subsequent microextrusion printing of a coating on its basis is effective in fabricating composite planar nanostructures with a uniform component distribution. This method is promising for the development of SOFC composite anodes of complex geometry, and the electrical conductivity of the material can also be improved by its additional high-temperature treatment.

## 4. Conclusions

The process of the hydrothermal synthesis of hierarchically organized nanomaterials with the core-shell structure of the composition ((CeO_2_)_0.8_(Sm_2_O_3_)_0.2_)@NiO was studied. Conditions were determined for the hydrothermal synthesis of (CeO_2_)_0.8_(Sm_2_O_3_)_0.2_ nanospheres of about 250 nm in size consisting of smaller particles (average size—10 ± 2 nm, average SCR size—8.0 ± 0.7 nm), and an approach to modifying their surface by growing the NiO shell with the formation of core-shell type structures evenly distributed between larger nickel(II) oxide nanosheets (less than 10 nm thick) was developed. When studying the microstructure of the modified oxide nanospheres, it was found that the NiO shell consisted of thin nanosheets and developed a hierarchically organized surface. It was shown that during the formation of the NiO shell, the average CSR size of (CeO_2_)_0.8_(Sm_2_O_3_)_0.2_ decreased by 36% due to hydrothermal treatment in the nickel-containing reaction system. The resulting nanopowder was used as a component of functional inks in the microextrusion printing of the corresponding composite coating. It was found that the obtained coating is characterized by high porosity, which is considered to be one of the most important characteristics of anodic coatings. At the same time, its microstructure fully corresponds to the composite powder used.

It was determined that the NiO nanosheets, between which the modified nanospheres were distributed, were highly porous (the average pore size was 8 ± 1 nm). The surface analysis of the obtained coating by the AFM methods confirmed that the nanospheres were covered by a NiO shell with a developed surface, and the larger NiO nanosheets were characterized by high porosity. The local electrophysical properties of the coating were characterized by KPFM. It was demonstrated that a shift in the charge carrier density in the intergranular region took place, and the determined value of the electronic work function of the nanospheres surface was about 4.698 eV. The nonlinear character of the current dependence on bias voltage was determined by this method in the whole investigated voltage range, which was determined by the presence of several rectifying contacts in the circuit. The obtained results may point to the presence of the rectifying contact associated with the p–n junction between the (CeO_2_)_0.8_(Sm_2_O_3_)_0.2_ core and the NiO shell. In addition, the temperature dependence of the specific conductivity of the studied material was determined, which had a linear character, and the conductivity increased by several orders of magnitude when the temperature increased from 50 to 600 °C. Thus, the proposed approach is promising for the creation of SOFC composite anodes of complex geometry.

## Data Availability

The data presented in this study are available in the article.
